# Preliminary Assessment of the Need and Awareness of Augmentative and Alternative Communication Systems in Armenia

**Published:** 2019-01

**Authors:** Tigran PETROSYAN, Hasmik MKRTCHYAN, Zvart HARUTYUNYAN, Armenuhi AVAGYAN

**Affiliations:** Department of Speech Therapy and Rehabilitation, Armenian State Pedagogical University after Khachatur Abovyan, Yerevan, Armenia

## Dear Editor-in-Chief

The goal of the study was to conduct a preliminary survey and determine the number of people who could benefit from augmentative alternative communication (AAC) in selected clinics of Armenia using an epidemiological approach. There is little epidemiological evidence available regarding the prevalence of need for AAC. The reviews of existing data in different countries rely on collating studies.

Figures used to estimate AAC need in different countries need to be reviewed on a regular basis because of increasing prevalence of children with disabilities surviving longer (1); the prevalence of individuals living with complex neurological conditions; longevity; the availability of sophisticated AAC strategies and equipment; and expectations of individuals and their families (2).

AAC encompasses a range of methods and techniques used by people who have impairments of speech, language, and communication. It includes technologies such as computerized systems and voice output communication aids and non-technological systems such as symbols and picture charts.

This research attempted to ask the following questions in order to determine the prevalence of people who could benefit from AAC: How many people are there with each of these medical impairments/disorders? How many of those people with the listed medical condition have speech, language and communication needs? How many of those people with speech, language and communication needs with the listed medical impairments/disorders could benefit from AAC?

A list of medical disorders of those people who could benefit from AAC was generated using a systematic literature review (3,4) and consultation with AAC professionals. The questionnaire used in the survey was adapted from a trial conducted in the UK (5). General practitioners - doctors working in 3 clinics of different cities in Armenia took part in the study. The survey tried to identify also the awareness of medical professionals about the AAC technologies.

There were 39 medical disorders identified through a systematic literature review. Table 1 shows that 169 people have one of these conditions. There are 104 people who have speech, language or communication needs to be associated with these conditions, and there are 54 people who could benefit from AAC. Only 3 from 24 medical professionals had information about AAC technologies and none of them have applied AAC in practice.

**Table 1:** Prevalence of augmentative alternative communication (AAC) need in selected population

***Have you heard of AAC technologies before***		***Yes (3)***	***No (21)***
***Have you applied AAC technologies for your patients***		***Yes (0)***	***No (24)***
***Diseases and Syndromes***	***Number of patients***	***Number of patients that have a speech problem***	***Number of patients withspeech problems that need AAC***
Stroke/CVA	16	10	6
Head/brain injury	14	9	6
Multiple sclerosis	6	3	1
Motor neuron disease			
Parkinson’s disease	17	13	8
Dementia/Alzheimer’s	28	15	10
Friedreich’s ataxia			
Multiple systems atrophy			
Head and neck cancer	4	3	3
Guillain–Barre syndrome	3	2	-
Cleft palate	7	6	-
Craniofacial abnormalities	3	2	-
Vision impairment Hearing impairment	12	9	2
Multisensory impairment			
PMLD Autistic spectrum disorder (ASD) Developmental delay	10	8	4
Other learning disabilities	12	10	6
Down syndrome	2	-	-
Angelman syndrome			
Huntington’s disease			
Prader–Willi			
Rett syndrome			
Williams syndrome			
Cerebral palsy	18	8	4
Specific language impairment			
Muscular dystrophy	3	1	-
Myasthenia gravis	2	-	-
Leigh’s disease			
Absence epilepsy			
Arthrogryposis (developmental non-progressive)			
Burns	2	1	1
Cerebellar ataxia			
Chromasomal mosaicism			
Meningo-encephalitis	4	2	1
Merrf syndrome			
Mitochondrial cytopathy			
Multi-systems atrophy			
Schizencephaly			
Vocal cord palsy	2	2	2
Voice disorder			
Total	169	104(61.5%)	54 (51.9%)

There is a large number of other more rare diseases for which we have insufficient information to arrive at a reliable estimate. People often have comorbidities, for example, cerebral palsy with learning disabilities. However, the data are for primary evaluation so this effect should be minimal.

Two conditions that represent 26.6% of the cohort consist of Alzheimer’s/dementia and Parkinson’s disease. The next sizeable cohort of people who could benefit from AAC were those with autistic spectrum conditions, learning disabilities, stroke/CVA, cerebral palsy, head/brain injury, profound, and multiple learning difficulties and motor neuron disease. 75.1% of people who could benefit from AAC have 8–10 conditions.

Current figures for use of AAC do not fully represent the national picture and broader study is required to get accurate estimates. No similar studies have been conducted in other countries of the region.
